# Synthesis and Electrical Property of Graphite Oxide-like Mesoporous *N*‑Carbon Derived from Polyimide-Covalent Organic Framework Templates

**DOI:** 10.1021/acsomega.5c03968

**Published:** 2025-08-22

**Authors:** Atsushi Nagai, Radian Febi Indrawan, Arthisree Devendran, Mozhgan Shahmirzaee, Sandhya Sharma, Hassan Alipour, Krzysztof Łyczko, Atsunori Matsuda

**Affiliations:** † Next-Generation Energy Systems Group, Centre of Excellence ENSEMBLE3 sp. z o.o., Wolczynska 133, Warsaw 01-919, Poland; ‡ Department of Electrical and Electronic Information Engineering, Toyohashi University of Technology, 1-1 Hibarigaoka, Tempaku-cho, Toyohashi, Aichi 441-8580, Japan; § Institute of Nuclear Chemistry and Technology, Dorodna 16, Warsaw 03-195, Poland

## Abstract

In this study, two PI-COFs, PI-TAPA-PMDI (twisted triphenylamine node) and PI-TAPB-PMDI (non-twisted triphenylbenzene node), were pelletized (∼10 mm diameter) under 90 MPa and carbonized at 600 °C in argon for 2–50 h. Carbonization produced nitrogen-doped, defective porous carbons with an enhanced electronic conductivity. Electrochemical impedance spectroscopy showed that PI-TAPB-PMDI COF-600 heated for 50 h had significantly lower resistance (*R*_s_ ≈ 14.14 Ω and *R*_ct_ ≈ 61.66 Ω) compared to shorter heating treatments (*R*_s_ ≈ 27.70 Ω), indicating improved electron transport and better interaction with a Fe­(CN)_6_^3–^/Fe­(CN)_6_^4–^ redox couple system. The XRD patterns verified the crystalline structure of PI-TAPA-PMDI and PI-TAPB-PMDI COFs, which reduces to an amorphous state during the carbonization progress. The XPS and FTIR results confirmed nitrogen incorporation and hydrogen bonding, while Raman and BET analyses revealed superior structural ordering and porosity in the PI-TAPB-PMDI COF compared to PI-TAPA-PMDI, respectively. For PI-TAPB-PMDI COF-600, increasing carbonization time raised BET surface area (up to 510 m^2^g^–1^) and promoted mesoporosity, with a pore size of 2.8 nm after 50 h treatment. In summary, PI-TAPB-PMDI COF-600 With a nitrogen content of 0.5% and conductivity of 3.02 × 10^–2^ S cm^–1^ demonstrates strong potential as a high-performance, functionalized graphite oxide-like material for energy storage applications.

## Graphical abstract

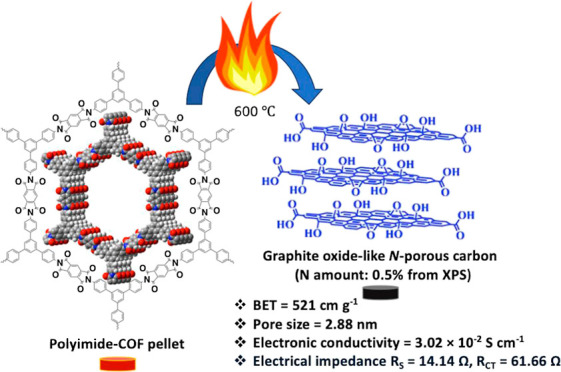

## Introduction

Whether the porous carbon is derived from natural sources or synthesized in the laboratory, the structural and surface properties of porous carbon depend largely on the physicochemical properties and synthesis route of the precursor. However, when conventional processes are used to produce porous carbon materials, precise control over the pore structure is often lacking. For example, carbonization and activation, which are widely used technological processes, result in the production of porous carbon with a polymodal pore size distribution (ranging from a few to 100 nm). Therefore, it is difficult to produce carbon with a predetermined porous structure, which requires the use of specialized approaches, resulting in higher costs. Among these approaches, template methods have been extensively studied due to their unique versatility and functionality.^1–4^

Therefore, porous carbon has attracted attention for its high specific surface area (*S*_BET_), good conductivity, and chemical and thermal stability, prompting the exploration of various practical applications. Examples include gas absorbers, oxygen reduction catalysts for fuel cells, and electrode materials for lithium-ion batteries as well as supercapacitors.^5–9^ Among the various methods for preparing porous carbon, template-assisted growth has been widely employed to produce a range of porous carbons, from microporous carbons using zeolite templates^10–13^ to structures with controlled pore size, morphology, and heteroatom configuration. This control is achieved by selecting an appropriate template, such as a metal oxide (combined with thermoplastic precursors)^2,14–16^ or a metal–organic framework (MOF).^17–19^ However, many of the template removal processes for forming pores in carbon materials still require the use of strong acids, such as hydrofluoric acid (HF) and hydrochloric acid (HCl), which are hazardous. As a result, simple template-based methods for carbon preparation have been extensively investigated.

In this study, we developed a facile method to prepare porous carbon by calcining only COFs. Generally, COFs are a new type of porous material composed of organic building blocks that form two-dimensional (2D) or three-dimensional (3D) polymer networks with precise porous structures based on covalent bonds.^20–25^ The covalent bonds of COFs can lead to high electrochemical durability and high resistance to acids or bases as compared to the coordination bonds of MOFs.^28–30^ COFs have been reported to be suitable for introducing new redox functional groups, unlike MOFs.^28–30^ Interestingly, similar to MOFs, the direct carbonization of COFs yields heteroatom-doped carbons, as COF structures naturally incorporate elements such as boron, nitrogen, and oxygen alongside carbon.^26,27^ Further, graphitized carbon materials have been obtained via the carbonization of COFs with π-conjugated structures.^25,28,31^ Tanaka et al. previously prepared porous carbon by direct carbonization of a boron-based COF with theoretical formula C_9_H_4_BO_2_ called COF-5 as an electrode material for supercapacitors.^32^ Recently, we reported the synthesis of porous carbon via pyrolysis of a polyimide-based COF (PI-COF)^9b^ at 700 °C, as well as the preparation of composites of PI-COF-700 with polyaniline (PANI) and poly­(3,4-ethylenedioxythiophene):poly­(styrenesulfonate) (PEDOT:PSS) for use as electrode materials in supercapacitor applications. The modified composite material exhibited a high specific capacitance value of 729.17 F g^–1^ at an applied current density of 2 A g^–1^.^9b^ The porous carbon derived from aromatic-imine-linked COFs exhibited excellent electrical properties and performance for capacitor applications. Aromatic polyimide (PI) is a unique material for carbonization and has attracted attention in the research field due to its easy processability, efficient graphitization, and high carbon yield. In particular, in laser-induced carbonization, PI substrates are mainly used because of the strong influence on the carbon skeleton (Kapton), which can obtain a rigid structure.^33–35^ However, the laser-induced process is so fast that the carbon purity in this procedure is rather low or insufficient.^36–38^ The unique molecular structure, chemical inertness, and thermal stability of aromatic polyimide (PI) materials should be strategically exploited. However, the conductivity and behavior of porous carbons, including carbon templating-synthesized from PI, have not been well-explored. Herein, this study investigates the porosity and conductivity of porous carbon synthesized via templating from polyimide-COFs and evaluates the electrical properties of the carbons prepared at high temperature (600 °C).

## Results and Discussion

Two polyimide-linked COFs (PI-COFs; PI-TAPB-PMDI COF and PI-TAPA-PMDI COF) were prepared from the condensation reaction of pyromellitic dianhydride (PMDA) with amines-substituted triphenylbenzene (TAPB) or -substituted triphenylamine (TAPA) under solvothermal conditions according to previous works (Figure 1A).^5,33a^ The characterization of the obtained COFs was studied by Fourier-transfer infrared (FT-IR) and Raman spectroscopies, nitrogen gas absorption, and XRD measurements.

**Figure 1:**
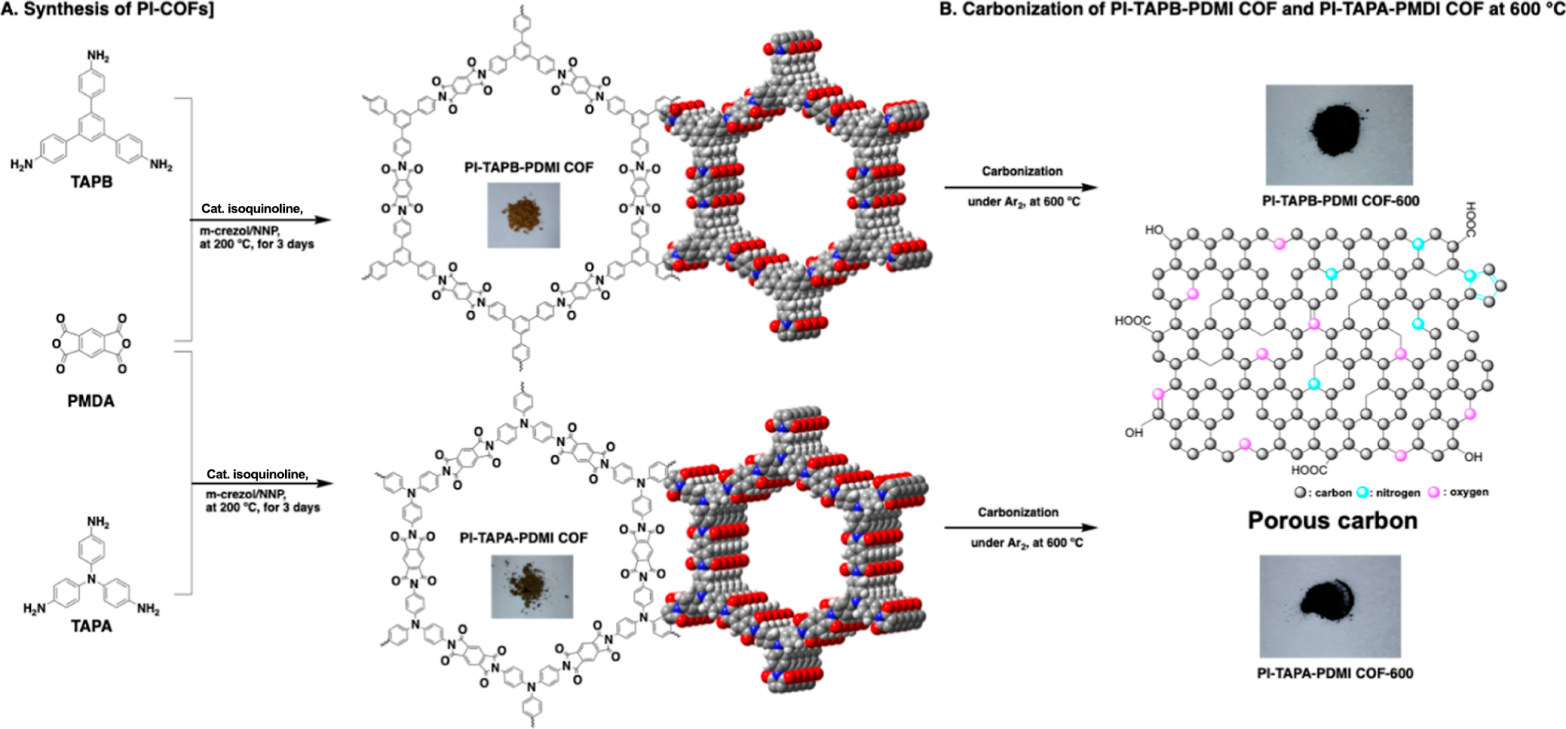
(A) Synthetic scheme of polyimide-COFs as the PI-TAPB-PDMI COF and PI-TAPA-PDMI COF, photographs of obtained COF solids, and DFT calculation of hexagonal pore and 4 layered structures^33^ and (B) the chemical structure and photographs of PI-TAPB-PDMI COF-600 and PI-TAPA-PDMI COF-600 after carbonization at 600 °C for 50h.

Thermogravimetric analysis (TGA) was performed on PI-TAPA-PMDI and PI-TAPB-PMDI COFs to evaluate their suitability for producing porous carbon PI-COFs, as shown in Figure S1. The results align with our previous study.^9b^ The carbonization temperature was previously selected as 700 °C, where the materials exhibit stability with minimal weight loss. However, in this study, a lower carbonization temperature of 600 °C was chosen. This temperature lies between the melting point and the initial decomposition temperature of the PI-TAPB-PMDI COF, allowing for a slower and more controlled carbonization process. Therefore, PI-TAPB-PMDI and PI-TAPA-PMDI COFs were processed into pellets of about 10 mm in diameter, pressed at 90 MPa, and heated under an argon atmosphere up to 600 °C at different hours. The final carbonized samples are displayed in Figure 1B. The behavior of PI-COFs was investigated from the relationship between transition, porosity, and electronic conductivity of pyrolyzed PI-COFs-600 (PI-TAPB-PMDI COF-600 and PI-TAPA-PMDI COF-600). The powders of the PI-TAPB-PMDI COF and PI-TAPA-PMDI COF were changed from light brown and dark brown, respectively, to darker shades (refer to Figure 1A).

The XRD patterns of PI-TAPB-PMDI COF and PI-TAPA-PMDI COF are shown in Figure 2A. Intense diffraction peaks at 2θ = 3.55°, 5.87°, 6.67°, and 12° of PI-TAPB-PMDI COF and 2θ = 3.35°, 5.81°, 6.66°, and 11.79° of PI-TAPA-PMDI, which were assigned to 100, 220, 310, and 400 facets, respectively, represent the ordered structure of the crystalline polyimide covalent organic framework (PI-COF). In addition, the interlayer π–π stacking of PI-TAPB-PMDI COF and PI-TAPA-PMDI COF was confirmed by respective small broad peaks at around 24° (001). These data result in the eclipsed AA stacking configuration before carbonization.^33^ As carbonization progresses (2, 5, 10, 15, 20, 25, and 50 h), the peaks gradually broaden and lose intensity, indicating a decrease in long-range order. This suggests that the COF structure begins to break down, leading to partial amorphization. At higher carbonization times (≥20 h), the characteristic COF peaks almost disappear, indicating that long-range periodicity is lost. In other words, the pyrolyzed PI-COFs transition from an ordered COF to a disordered carbonaceous phase.^39^ In addition, the crystal structures of PI-TAPA-PMDI COF and PI-TAPB-PMDI COF, obtained through DFT simulations, are illustrated in Figure 2B and the corresponding crystallographic data for their unit cells are provided in Tables S1 and S2.

**Figure 2:**
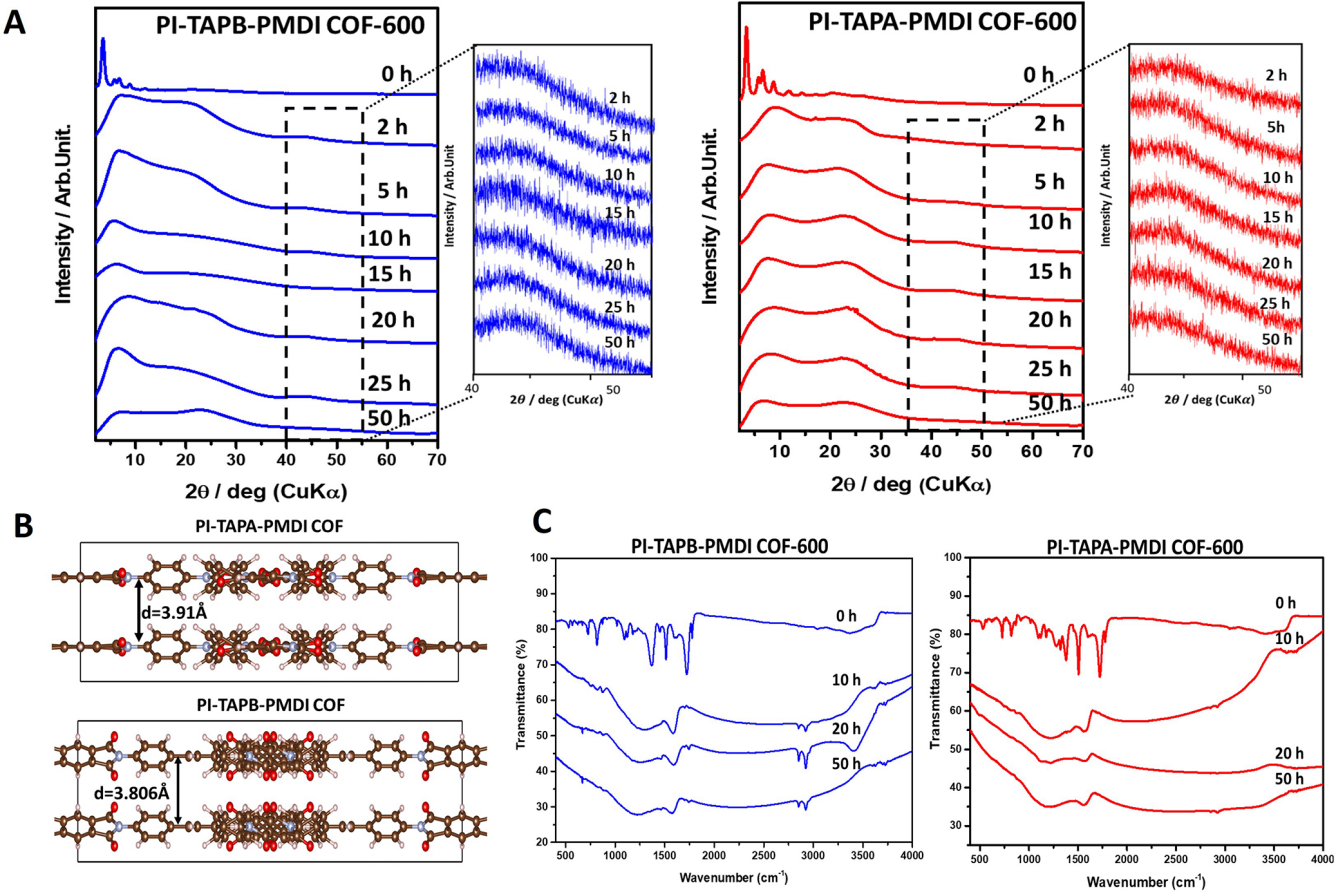
(A) XRD patterns of the PI-TAPB-PMDI COF and PI-TAPA-PMDI COF and carbonized to 600 °C at various times (0, 2, 5, 10, 15, 20, 25, and 50 h), (B) AA stacking two layered structures by VASP and DFT calculation and (C) FT-IR spectra of PI-TAPB-PMDI COF-600 and PI-TAPA-PMDI COF-600 carbonized at selected times (0, 10, 20, and 50 h).

The higher stability of the PI-TAPB-PMDI COF compared to the PI-TAPA-PMDI COF can be interpreted in terms of intermolecular interactions and stacking energy, which PI-TAPB-PMDI and PI-TAPA-PMDI COFs showed to be 34.177 and 21.360 eV, respectively. These energy amounts were calculated based on the single A stacked energy and double AA stacked energy for the PI-TAPB-PMDI COF (A stacked energy: −940.145 and AA stacked energy: −905.968 eV) and PI-TAPA-PMDI COF (A stacked energy: −851.622 and AA stacked energy: −830.262 eV), respectively. In addition, the stacking layer distance of PI-TAPB-PMDI with 3.806 Å was calculated to be less than PI-TAPA-PMDI with 3.910 Å. As shown in Figure 2B, the TAPB linker has a more rigid, planar, and extended π-conjugated system as compared to the TAPA. This enhances the π–π stacking interactions between the COF layers, resulting in better ordered stacking and higher crystallinity. In addition, the PI-TAPA-PMDI COF possesses a triphenyl-substituted nitrogen (N) atom, which means that it is more easily broken by lone pairs of electrons at high temperature, which can disrupt conjugation within the molecular structure.

Figure 2C displays the carbonization time-dependent Fourier transform infrared (FT-IR) absorption measurement of PI-COFs heated to 600 °C at selected times (0, 10, 20, and 50 h). IR spectra show absorptions at 1773 and 1776 cm^–1^ for the PI-TAPB-PMDI COF and 1772 and 1775 cm^–1^ for the PI-TAPA-PMDI COF, corresponding to asymmetric and symmetric vibrations of the C=O groups of the five-membered imide rings, respectively, while peaks at 1366 cm^–1^ for PI-TAPB-PMDI and 1379 cm^–1^ for the PI-TAPA-PMDI COF are attributed to the stretching vibration of the C–N–C moiety. As observed, the intensity of the C=O vibration peaks in the imide rings decreases while the C=N stretching vibrations appears at 1321 cm^–1^, indicating the formation of an iso-imide bond as the derivative progresses in the reaction.^40,41^ The peak at 1506 cm^–1^ is attributed to aromatic C=C bonds. As the carbonization reaction time increases, the characteristic peaks of C=N and C=O gradually decrease, and the characteristic peak of the aromatic C=C intensively increases, indicating that the carbonization of PI-COFs becomes graphitic with an increasing reaction time.

Figure 3A represents the Raman spectra of PI-COFs heated to 600 °C at different times from 2 to 50 h. As can be seen from the figures, during the heating process, there is a slight variation in the structural disordered (D band) and ordered sites (G band).^42^ The *I*_D_/*I*_G_ ratio represents the intensity ratio calculated from the D band (defective sites, sp^3^-hybridized carbon) to the G band (graphitic order, sp^2^-hybridized carbon). For the case of PI-TAPA-PMDI COF-600 (red curves in Figure 3A), the *I*_D_/*I*_G_ intensity ratio value starts from 0.86 (for 2 h) and increases to 0.99 (for 20 h), showing that the disorder in the structure gradually increases. After 20 h of heating, there was a visible fluctuation with the intensity ratio values decreasing slightly to 0.9646 (at 25 h) and 0.98 (at 50 h). This suggests that the structural degradations are initially dominant, but after prolonged heating, the material may have reached a stable disordered phase. Similarly, for the case of PI-TAPB-PMDI COF-600 (blue curve in Figure 3A), the *I*_D_/*I*_G_ intensity ratio value starts from 0.92 (2 h) and reaches 1.06 (for 15 h), showing a stable increase in the structural disorder at higher temperature. After 15 h, the intensity ratio value decreased significantly to 0.91 (for 20 h) but remained relatively higher, up to 0.97 (for 50 h). This observation suggests that the PI-TAPB-PMDI COF undergoes a more pronounced transformation during the heating process than the PI-TAPA-PMDI COF, indicating more persistent structural defects in the PI-TAPB-PMDI COF system. In particular, the maximum *I*_D_/*I*_G_ ratio value obtained for the PI-TAPB-PMDI COF ∼1.06 is higher than the PI-TAPA-PMDI COF ∼0.99, indicating that the PI-TAPB-PMDI COF undergoes greater structural disorder. These defects and irregular structures can introduce additional chemically active sites, facilitating improved electrical and electrochemical performance of carbon materials.^43^ This observation is consistent with XRD results showing an amorphous carbon-like structure for the PI-TAPA-PMDI COF and PI-TAPB-PMDI COF at higher heating temperatures. However, both COFs exhibit similar *I*_D_/*I*_G_ ratios (∼0.98 to 0.97) after prolonged heating up to 50 h, indicating that the degree of disorder and structural alteration is similar at the end.

**Figure 3:**
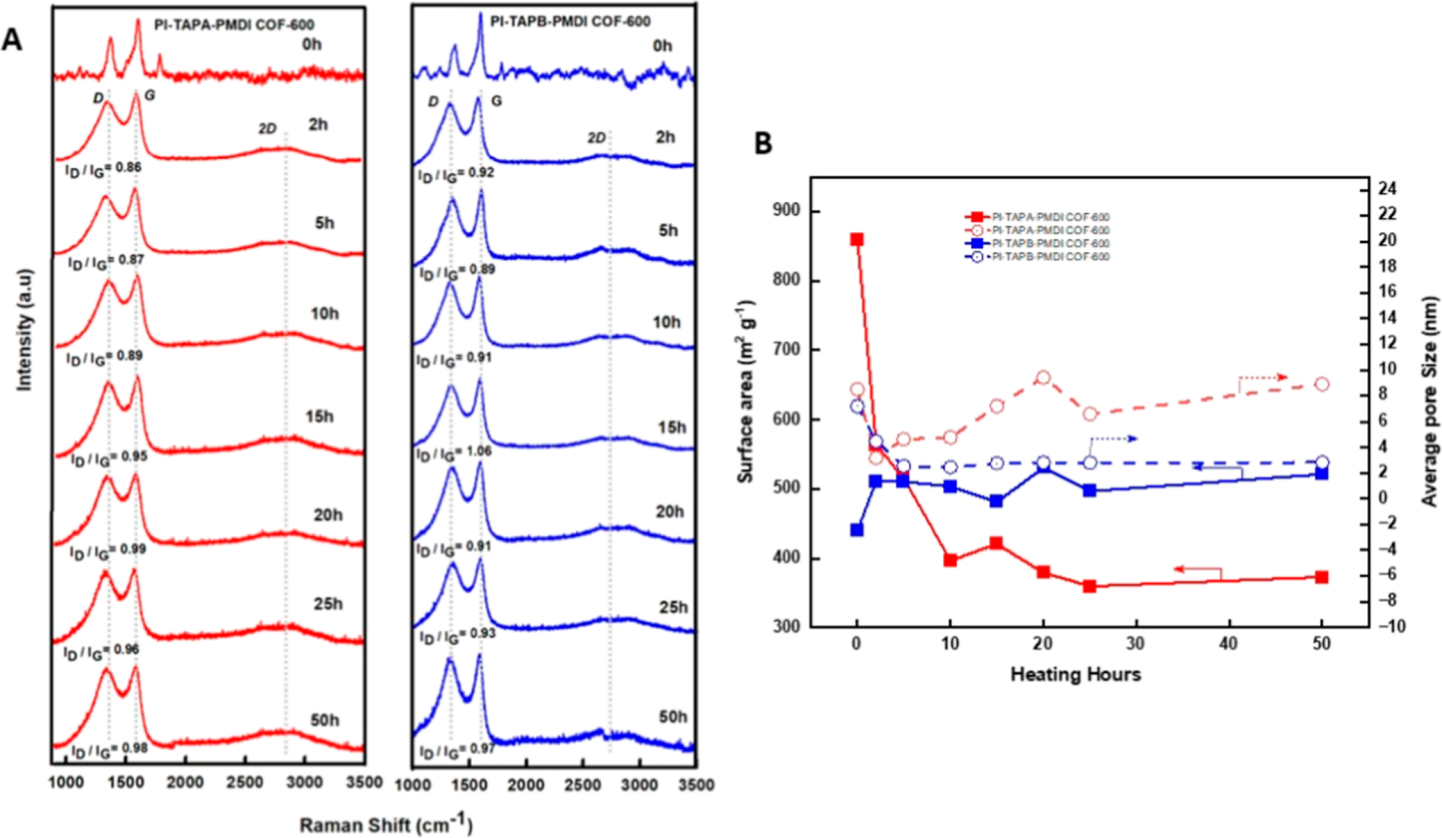
(A) Raman spectroscopy of two different porous carbons from PI-TAPA-PMDI COF-600 and PI-TAPB-PMDI COF-600 at various heating times (from 2 to 50 h) and (B) the relationship between BET surface areas and average pore sizes of PI-TAPA-PMDI COF-600 and PI-TAPB-PMDI COF-600.

To investigate the dependence of PI-COFs on carbonization time, nitrogen gas adsorption/desorption analysis was conducted to assess the stability of porosity, as shown in Figure 3B. All of the Brunauer–Emmett–Teller (BET) test samples followed the nitrogen type IV isotherms method. The average pore sizes were determined using the Barrett–Joyner–Halenda (BJH) method. Accordingly, the BET surface areas and average pore sizes of the PI-TAPA-PMDI COF and PI-TAPB-PMDI COF were 859.95 and 440.06 m^2^ g^–1^ and 8.50 and 7.27 nm, respectively.

All nitrogen isotherms and BJH pore size distributions versus carbonization time are shown in Figures S2–S5. For PI-TAPA-PMDI COF-600, increasing the carbonization time at 600 °C leads to a gradual decrease in the BET surface area and an increase in the pore size. This trend corresponds to the formation of more disordered carbon structures, as indicated by Raman spectroscopy. In contrast to this observation, PI-TAPB-PMDI COF-600 shows a relatively stable *I*_D_/*I*_G_ ratio after an initial rise, indicating more thermally stable graphitic domains. Correspondingly, its BET surface area increases with time, while the pore size initially decreases up to 5 h and then stabilizes with further carbonization time. In Figures S1c and S2g, the observed hysteresis, where the desorption curve falls below the adsorption curve, may be attributed to several factors. Such behavior can result from kinetic limitations, delayed capillary evaporation, or irreversible adsorption processes. Additionally, in some cases, framework flexibility or partial pore collapse during the measurement may also contribute to these deviations.

Previous studies have reported that the graphitization of aromatic polyimides proceeds via the formation of an isoimide structure. This transformation is driven by a high-temperature equilibrium reaction (typically at 500–600 °C)^40,41^ between the imide and isoimide forms. At these temperatures, the conversion from imide to isoimide becomes effectively irreversible.^44^ Importantly, in both studies, the polymer backbone remains intact and is not cleaved during the heating process. Based on these findings, it can be inferred that graphitization at elevated temperatures leads to an increase rather than a decrease in the surface area of polyimides. Therefore, in this study, the PI-TAPA-PMDI COF contains triphenylamine moieties as twisted nodes within its 2D layered polymer structure. During carbonization up to 25 h, random degradation occurs primarily at these twisted node sites, leading to a decrease in BET surface area and irregular changes in pore size. In contrast, the PI-TAPB-PMDI COF, which features triphenyl benzene moieties as planar, non-twisted nodes, demonstrates greater structural stability, suggesting it is a more suitable precursor for graphitization. Finally, the PI-TAPB-PMDI COF carbonized at 600 °C for 50 h had 521.11 m^2^ g^–1^ as a BET surface area and an average pore size of 2.86 nm. In contrast, PI-TAPA-PMDI COF-600 had a 372.60 m^2^ g^–1^ BET surface area and 8.90 nm particle size. The overall pore architecture of the carbonized COFs ranges from 2 to 5 nm, representing aggregated particles during the heating process.

The morphology of PI-COFs and PI-COFs-600 pyrolyzed for 50 h was examined using scanning electron microscopy (SEM) at an accelerating voltage of 10 kV. SEM images of the PI-TAPA-PMDI COF and PI-TAPB-PMDI COF precursors (Figure 4A,C) show agglomerated particles with average sizes of 259 and 83.97 nm, respectively. After carbonization, the resulting porous carbon materials exhibit a loosely packed structure (Figure 4B,D). Most samples display a surface morphology characterized by irregularly stacked, block-like structures. Compared to the precursors, the carbonized samples show a reduced particle size, approximately 62.85 and 42.1 nm (Figure 4C,D); this size reduction is likely associated with thermal decomposition and structural shrinkage during pyrolysis, potentially accompanied by the formation of pores, as volatile components are eliminated. This may lead to structural densification or aggregation and potential rearrangement into more thermally stable domains.

**Figure 4:**
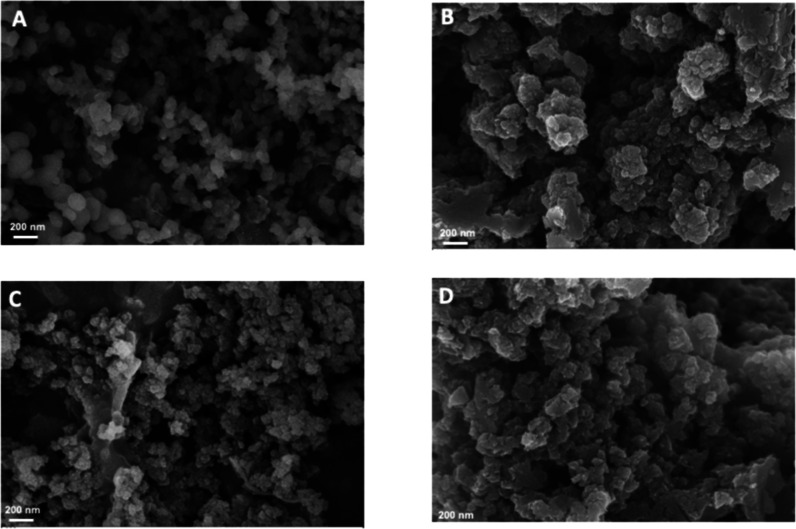
SEM images of (A) PI-TAPA-PMDI COF, (B) PI-TAPA-PMDI COF-600 for 50 h, (C) PI-TAPB-PMDI COF, and (D) PI-PAPB-PMDI COF-600 for 50 h.

Figure 5A shows the relationship between the electrical conductivities and the BET surface areas for PI-TAPA-PMDI COF-600 and PI-TAPB-PMDI COF-600 for 50 h, respectively. Regardless of whether the BET surface area increases or decreases, longer carbonization times result in higher electrical conductivity. This observation suggests that electrical conductivity is not directly dependent on the surface area.

**Figure 5:**
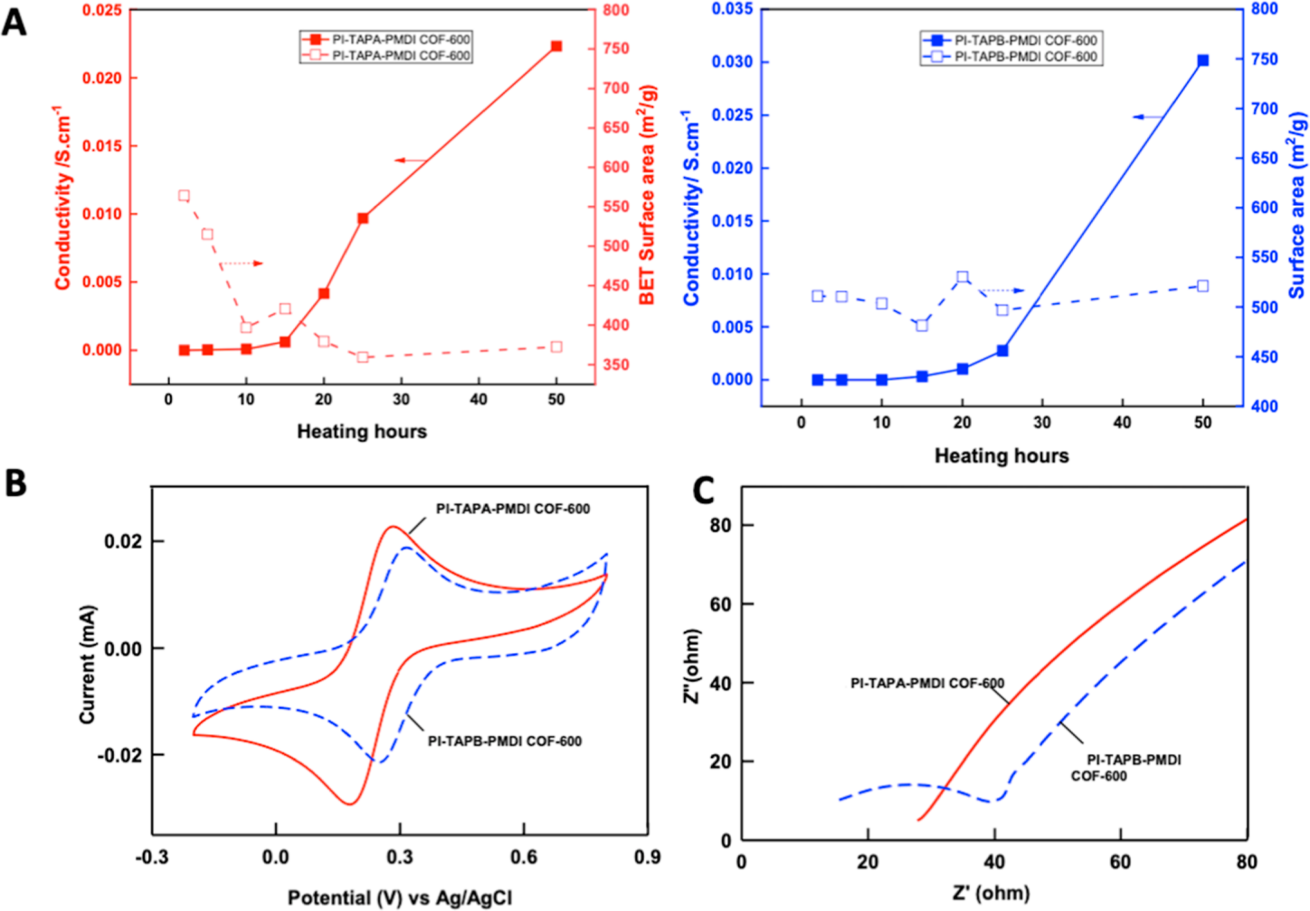
(A) Respective relationship between electronic conductivities and BET surface areas of PI-TAPA-PMDI-COF-600 for 50 h and PI-TAPB-PMDI-COF-600 for 50 h, (B) CV responses of PI-TAPA-PMDI-COF-600 for 50 h and PI-TAPB-PMDI-COF-600 for 50 h, and (C) Nyquist plots of PI-TAPA-PMDI-COF-600 for 50 h and PI-TAPB-PMDI-COF-600 for 50 h.

The electrochemical characterization of two different carbonized imide COFs was conducted using the CV technique with 5 mM Fe­(CN)_6_^3–^ in a 0.5 M KCl solution electrolyte medium (Figure 5B). As can be seen, both curves exhibit similar redox peaks at *E*° = 0.3 V vs Ag/AgCl, which is characteristic of the Fe­(CN)_6_^3–^ redox coupled reaction. Notably, PI-TAPA-PMDI COF-600 for 50 h has the higher peak current appearance compared to the porous carbon of PI-TAPB-PMDI COF-600 for 50 h, suggesting more efficient electron transfer at the electrode/electrolyte interface of the former system. Further analysis via electrochemical impedance spectroscopy (EIS) exhibits a straight line suggesting a diffusion-controlled high charge transfer resistance for Figure 5C PI-TAPA-PMDI COF-600 for 50 h, whereas PI-TAPB-PMDI COF-600 for 50 h showed a semicircular pattern followed by a linear straight line in the low-frequency region for the ferricyanide system. This observation implies that PI-TAPB-PMDI COF-600 has a more efficient charge transfer with low diffusion limitations. The curve fitting was done using the Randles equivalent circuit model [*R*_s_(*R*_ct_ *W*)*C*_dl_], where *R*_s_ represents the equivalent series resistance (intercept of the semicircle), *R*_ct_ is the charge transfer resistance (the semicircular diameter region), *C*_dl_ is the double layer capacitance, and *W* is the Warburg diffusion resistance (straight line in the low-frequency region), respectively.^45^ Using in-built software, the individual circuit components were extracted. From these observations, PI-TAPA-PMDI COF-600 for 50 h has a high resistance of *R*_s_ ∼27.70 Ω and may have reduced availability of active sites inhibiting the impedance response. However, the PI-TAPB-PMDI COF-600 for 50 h indicates better charge transfer behavior with a low *R*_s_ value ∼14.14 Ω and *R*_ct_ 61.66 Ω. These low resistance values suggest more efficient interaction with the Fe­(CN)_6_^3–^/Fe (CN)_6_^4–^ redox system via a one-electron transfer reaction potentially contributing to enhanced electrical characteristics of the PI-TAPB-PMDI COF-600 system. The improved structural properties of PI-TAPB-PMDI COF-600 after 50 h likely facilitate the charge transport pathways. This observation is supported by FTIR analysis, indicating hydrogen bonding, and XPS results showing N-doping, which together may account for the enhanced electrical performance.

Since the PI-TAPB-PMDI COF precursor is the optimal choice for porous carbon synthesis due to its higher intensity ratio observed in Raman analysis and superior surface properties from BET analysis as compared to the PI-TAPA-PMDI COF precursor, the surface chemistry of PI-TAPB-PMDI COF-600 was analyzed by XPS peak assignments in Figures S6 and S7 and Tables S3 and S4. From C 1s, N 1s, and O 1s spectra in Figure S6, the C 1s peak can be assigned to C–C at 284.6 eV, which represents the aromatic rings contributing to 82.6% of the atomic composition. 285.54 eV contributes to the C–O and C–N, which supports the presence of the imide group. The peaks at 288.82 and 291.04 eV are assigned to C=O, COOH corresponding to the carbonyl group from the aromatic system. Followingly, the nitrogen (N 1s) peaks at 400.7 eV and 402.2 eV are ascribed to C–NH^+^ protonated nitrogen species and C–N & N=C are the nitrogen groups present in the imide ring contributing to 6.4% atomic composition. Finally, the oxygen O 1s peaks at 532.3 and 533.69 eV correspond to C=O of the imide group and C–O aromatic oxygen present within the conjugated systems (contributing to 11.0% atomic compositions). The C–O aliphatic peak at 535.12 eV corresponds to the hydroxyl group.^46^ Hence, the PI-TAPB-PMDI COF exhibits a well-defined electronic structure, making it a promising precursor for carbonizing. After heating the PI-TAPB-PMDI COF to 600 °C (as depicted in Figure S7 and Table S4), the porous carbon has five major C 1s peaks at 283.1, 284.6, 286.2, 288, and 289.6 eV which are attributed to sp^2^, sp^3^, C–O and C–N, C=O, and COOH with a major contribution of 95% atomic composition, respectively. While N 1s also showed three peaks at 397, 399.5, 402.5 eV corresponding to C–N and N=C which is pyridinic nitrogen (present in graphitic edge) with 0.5% atomic composition, N–C=O the pyrrolic nitrogen (present in the imide group) and C–NH^+^, which represents the existence of graphitic nitrogen, respectively.^47^ Similarly, O 1s has three peaks corresponding to C=O, C–O (with 3.7% atomic composition), and the aromatic C–O at 530.8, 532.4, and 534.0 eV, respectively. The marked shift from the N 1s spectra may be due to the conversion of the nitrogen group in the imide ring into more aromatic nitrogen resulting in a nitrogen-doped defective porous carbon structure. These observations align well with the FTIR spectra; additionally, the 0.5% atomic composition of the nitrogen linkage in the porous carbon structure provides more active sites for enhanced electrical conductivity.^48^ The XPS results reveal a significant increase in sp^2^-hybridized C–C bonding and a reduction of oxygen-containing functional groups. Additionally, the persistent nitrogen functionalities suggest enhanced electronic delocalization and stabilization of the conjugated networks. These bond level transformations are not captured by the Raman *I*_D_/*I*_G_ ratio but, likely contribute to the sharp increase in conductivity after 50 h heating treatment, supporting the structural reorganization and conjugation enhancement beyond 20 h.

## Summary

Two PI-COFs as PI-TAPB-PMDI and PI-TAPA-PMDI COFs were prepared from the condensation reaction of pyromellitic dianhydride (PMDA) with 1,3,5-tri­(4-aminophenyl)­benzene (TAPB) or tris­(4-aminophenyl)­amine (TAPA) under solvothermal conditions. We investigated a facile and unique process for preparing nitrogen-doped porous carbon by direct carbonization of two polyimide-COFs (PI-COFs) acting as self-templates at 600 °C. We examined the relationships among surface area, pore size, electrical conductivity, BET surface area, and electrochemical properties such as impedance and resistance. Regardless of changes in the BET specific surface area, the electronic conductivity gradually increases with longer carbonization times. PI-TAPA-PMDI COF-600 carbonized for 50 h exhibits a high resistance (*R*_s_ ∼27.70 Ω), which may indicate a reduced number of active sites, thereby limiting its impedance response. However, the PI-TAPB-PMDI COF-600 for 50 h indicates more efficient electron transfer behavior with a low *R*_s_ value ∼14.14 Ω and *R*_ct_ 61.66 Ω. XPS data showed increased sp^2^ C–C bonding and the persistence of nitrogen-containing functional groups, including graphitic and pyrrolic nitrogen, which promotes the charge delocalization and conductivity. As a result, the porous carbon with few nitrogen atoms (N atom; 0.5%, conductivity of 3.02 × 10^–2^ S cm^–1^, BET of 510 m^2^ g^–1^, and pore size of 2.80 nm) carbonized for 50 h showed the best functionalized graphite oxide-like structure highlighting the potential of PI-TAPB-PMDI COF as efficient an precursor for high-performance materials suitable for energy storage applications.

## Experimental Methods

### Materials

The PI-TAPB-PMDI COF and PI-TAPA-PMDI COF were prepared according to previous work.^9b,33^ Unless stated otherwise, all other reagents were obtained from commercial sources and used without further purification.

### Synthesis of Polyimide COFs (PI-TAPB-PMDI and PI-TAPA-PMDI COFs)

A 10 mL Pyrex tube was loaded with either 1,3,5-tri­(4-aminophenyl)­benzene (TAPB; 0.70 g, 2 mmol) or tris­(4-aminophenyl)­amine (TAPA; 0.58 g, 2 mmol), along with PMDA (0.65 g, 3 mmol), in a mixture of 10 mL of *m*-cresol and 10 mL of *N*-methyl-2-pyrrolidone (NMP), with 0.18 mL of isoquinoline as an additive. The tube underwent degassing through freeze-drying at 77 K, was flame-sealed, and was subsequently heated at 200 °C for 5 days. After the reaction, the resulting solid was collected, washed three times with methanol and acetone, and separated via centrifugation. Further purification was performed using Soxhlet extraction in tetrahydrofuran (THF) for 24 h, followed by vacuum drying at 80 °C for 12 h. This process yielded the PI-TAPB-PMDI COF and PI-TAPA-PMDI COF as light-brown and dark-brown powders, respectively, with isolated yields of 88.8% (1.21 g) and 91.9% (1.13 g).

### Preparation of PI-TAPB-PMDI COF-600 and PI-TAPA-PMDI COF-600

After being pelletized (13 mm in diameter, 2 mm in thickness), the PI-COF was placed in an alumina crucible and heated form 2-50 h at a rate of 10 °C min^–1^ in a tubular furnace up to 600 °C under Ar pressure. Ultimately, PI-TAPA-PMDI and PI-TAPB-PMDI COFs were produced from the carbonized pellets, which had dimensions of 10 mm in diameter, 2 mm in thickness, and 197.4 mg per pellet. Figure 1 displays the PI-COF both before and after carbonization.

The thermal decomposition curves were recorded using a TA Instruments SDT Q600 thermogravimetry analyzer under a nitrogen atmosphere with a heating rate of 10 °C min^–1^. The Rigaku SmartLab 3 kW diffractometer (Rigaku Corporation, Tokyo, Japan) using Cu Kα radiation and a high-speed 1D silicon strip detector D/teX Ultra 250 were used to study X-ray powder diffraction. In reflection Bragg–Brentano geometry (θ/2θ scanning mode), the powder diffraction patterns were observed in the angular range 2° or with a scanning step of 0.01° and a speed duration time of 1° min^–1^.

IR spectra were registered in the 4000–650 cm^–1^ range on a Thermo Scientific Nicolet iS10 FT-IR spectrometer using a PIKE Technologies MIRacle accessory with a ZnSe crystal designed for the single-reflection horizontal ATR technique. i-Raman Plus, a portable equipment with BWIQ for analysis and BWID identification software, was used to perform Raman spectroscopy analysis. With a heating rate of 10 K min^–1^, TGA measurements were carried out using a NETZSCH STA 449 F1 Jupiter. Using a Brunauer–Emmett–Teller (BET) surface analyzer (3P, Micro 200), the samples’ surface area was examined. Prior to measurement, the samples were degassed in vacuum at 150 °C for one hour in order to eliminate any remaining water. The FEI QUANTA 250 FEG device was used to perform the FE-SEM.

The plane-wave Vienna ab initio simulation package’s implementation of DFT calculations was used.^49^ The core–electron interactions were investigated using the projector augmented wave approach, and the generalized gradient approximation of Perdew–Burke–Ernzerhof^50^ was chosen.^51^ Total energies for hexagonal unit-cell configurations of single-layer and AA-stacked structures were calculated to calculate the stacking energy of the AA-stacked structures. In all instances, single-point energies were obtained from self-consistent calculations with a cutoff energy of 520 eV and an energy convergence criterion of 10^–5^ eV.

A potentiostat (1280C, Solartron) was used to monitor DC polarization in order to calculate electrical conductivity. Pellets were exposed to voltages of 0.1, 0.15, 0.2, 0.25, and 0.3 V for 30 min at room temperature to perform the tests. At room temperature, around 80 mg of sample powder was uniaxially pressed into disks of about 10 mm in diameter at a pressure of 90 MPa to create the pellets.

## Supplementary Material



## Notes

Crystallographic data for the structures of PI-TAPA-PMDI COF and PI-TAPB-PMDI COF unit cells; TGA of PI-TAPA-PMDA-COF and PI-TAPB-PMDA-COF; BET surface area measurements before and after carbonization of PI-TAPA-PMDA-COF and PI-TAPB-PMDA-COF; BJH adsorption pore size distribution plots (d*V*/d*D*) before and after carbonization of PI-TAPA-PMDA-COF and PI-TAPB-PMDA-COF; XPS spectra of the PI-TAPB-PMDI COF and its peak assignments table; and XPS spectra of the carbonized PI-TAPB-PMDI COF 600 °C for 50 h and its peak assignments table (PDF)
