# Supplementary material for: Synthesis and Electrical Property of Graphite Oxide-like Mesoporous N‑Carbon Derived from Polyimide-Covalent Organic Framework Templates
Source: ACS Omega. 2025 Aug 22;10(35):39841–9. doi: 10.1021/acsomega.5c03968 (PMC12423787; doi:10.1021/acsomega.5c03968)
Supplement: Supplementary file 1 [file ao5c03968_si_001.pdf]

Supporting Information

Synthesis and electrical property of graphite oxide-like mesoporous *N*-carbon derived from polyimide-covalent organic framework templates

*Atsushi Nagai, *† Radian Febi Indrawan, ‡ Arthisree Devendran, † Mozhgan Shahmirzaee, † Sandhya Sharma, † Hassan Alipour, † Krzysztof Łyczko, § Atsunori Matsuda ‡*

[†] Next-Generation Energy Systems group, Centre of Excellence ENSEMBLE3 sp. z o.o., Wolczynska 133, Warsaw 01-919, Poland

[‡] Department of Electrical and Electronic Information Engineering, Toyohashi University of Technology, 1-1 Hibarigaoka, Tempaku-cho, Toyohashi, Aichi 441-8580, Japan

[§] Institute of Nuclear Chemistry and Technology, Dorodna 16, Warsaw 03-195, Poland.

*E-mail: atsushi.nagai@ensemble3.eu

Supplementary Figures:

Figure S1. TGA results of a. PI-TAPA-PMDA-COF, b. PI-TAPB-PMDA-COF
Figure S2. Nitrogen adsorption/desorption isotherms of a. PI-TAPA-PMDI COF, b. PI-TAPA-MDI COF-600 for 2h, c. PI-TAPA-PMDI COF-600 for 5h, d. PI-TAPA-PMDI COF-600 for 10h, e. PI-TAPA-PMDI COF-600 for 15h, f. PI-TAPA-PMDI COF-600 for 20h, g. PI-TAPA-PMDI COF-600 for 25h, h. PI-TAPA-PMDI COF-600 for 50h.
Figure S3. Nitrogen adsorption/desorption isotherms of a. PI-TAPB-PMDI COF, b. PI-TAPB-PMDI COF-600 for 2h, c. PI-TAPB-PMDI COF-600 for 5h, d. PI-TAPB-PMDI COF-600 for 10h, e. PI-TAPB-PMDI COF-600 for 15h, f. PI-TAPB-PMDI COF-600 for 20h, g. PI-TAPB-PMDI COF-600 for 25h, h. PI-TAPB-PMDI COF-600 for 50h.
Figure S4. BJH Adsorption Pore Size Distribution Plots (dV/dD) of a. PI-TAPA-PMDI COF, b. PI-TAPA-PMDI COF-600°C for 2h, c. PI-TAPA-PMDI COF-600°C for 5h, d. PI-TAPA-PMDI COF-600°C for 10h, e. PI-TAPA-PMDI COF-600°C for 15h, f. PI-TAPA-PMDI COF-600°C for 20h, g. PI-TAPA-PMDI COF-600°C for 25h, h. PI-TAPA-PMDI COF-600°C for 50h.
Figure S5. BJH Adsorption Pore Size Distribution Plots (dV/dD) of a. PI-TAPB-PMDI COF, b. PI-TAPB-PMDI COF-600°C for 2h, c. PI-TAPB-PMDI COF-600°C for 5h, d. PI-TAPB-PMDI COF-600°C for 10h, e. PI-TAPB-PMDI COF-600°C for 15h, f. PI-TAPB-PMDI COF-600°C for 20h, g. PI-TAPB-PMDI COF-600°C for 25h, h. PI-TAPB-PMDI COF-600°C for 50h.
Figure S6. XPS Spectra of PI-TAPB-PMDI COF
Figure S7. XPS Spectra of PI-TAPB-PMDI COF 600°C for 50h

Supplementary Tables:

Table S1. Crystallographic data for the structures of PI-TAPA-PMDI COF unit cell.
Table S2. Crystallographic data for the structures of PI-TAPB-PMDI COF unit cell.
Table S3. XPS peak assignments for PI-TAPB-PMDI COF
Table S4. XPS peak assignments for PI-TAPB-PMDA COF 600°C for 50h

Supporting Information

Table S1. Crystallographic data for the structures of PI-TAPA-PMDI COF unit cell.

Label	atom	x	y	Z	type	Occupancy
C2	C	0.04321	0.49928	0.00512	Uiso	1.00
C4	C	0.09520	0.51034	0.02455	Uiso	1.00
C7	C	0.34010	0.59425	0.15882	Uiso	1.00
C8	C	0.36539	0.56843	0.15908	Uiso	1.00
O1	O	0.11009	0.48141	0.06343	Uiso	1.00
H2	H	0.30458	0.57806	0.28542	Uiso	1.00
H3	H	0.34956	0.53304	0.28824	Uiso	1.00
C1	C	-0.00000	0.54628	0.00000	Uiso	1.00
H1	H	-0.00000	0.58103	-0.00000	Uiso	1.00
C6	C	0.28096	0.64048	-0.00000	Uiso	1.00
N1	N	0.12482	0.56241	-0.00000	Uiso	1.00
C9	C	0.41147	0.58853	-0.00000	Uiso	1.00
N7	N	0.33333	0.66667	0.00000	Uiso	1.00

s.g.	P622 (177)
a (Å)	31.369
b (Å)	31.369
c (Å)	3.910
alpha (°)	90.00
beta (°)	900.00
gamma (°)	120.00

Table S2. Crystallographic data for the structures of PI-TAPB-PMDI COF unit cell.

Label	atom	x	y	Z	type	Occupancy
O1	O	0.49067	0.09519	0.22388	Uiso	1.00
C4	C	0.21852	0.63890	0.35751	Uiso	1.00
C5	C	0.17441	0.61713	0.35733	Uiso	1.00
C49	C	0.42990	0.51204	0.63350	Uiso	1.00
C50	C	0.46381	0.50105	0.57599	Uiso	1.00
H2	H	0.23528	0.67003	0.23281	Uiso	1.00
H3	H	0.15690	0.63099	0.23796	Uiso	1.00
C1	C	0.31127	0.68873	0.50000	Uiso	1.00
H1	H	0.29398	0.70602	0.50000	Uiso	1.00
C2	C	0.28849	0.64424	0.50000	Uiso	1.00
C6	C	0.15253	0.57627	0.50000	Uiso	1.00
C3	C	0.37924	0.75847	0.50000	Uiso	1.00

N1	N	0.44632	0.89264	0.50000	Uiso	1.00
C53	C	0.53640	0.53640	0.34305	Uiso	1.00
H31	H	0.56374	0.56374	0.22544	Uiso	1.00

s.g.	P-31M (162)
a (Å)	36.5283
b (Å)	36.5283
c (Å)	3.8065
alpha (°)	90.0000
beta (°)	90.0000
gamma (°)	120.0000

a.

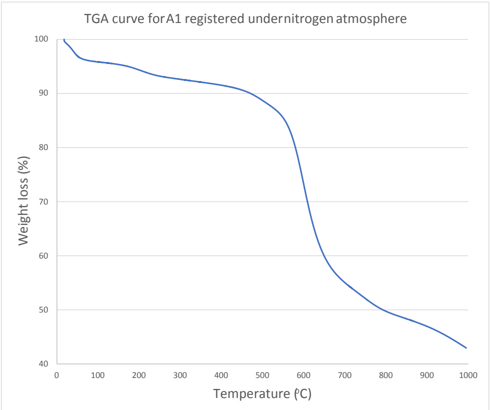

b.

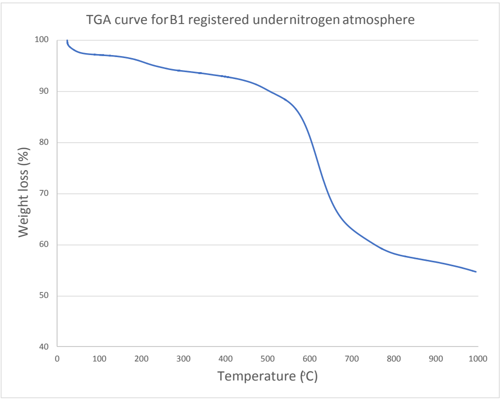

Figure S1. TGA results of a. PI-TAPA-PMDA-COF, b. PI-TAPB-PMDA-COF

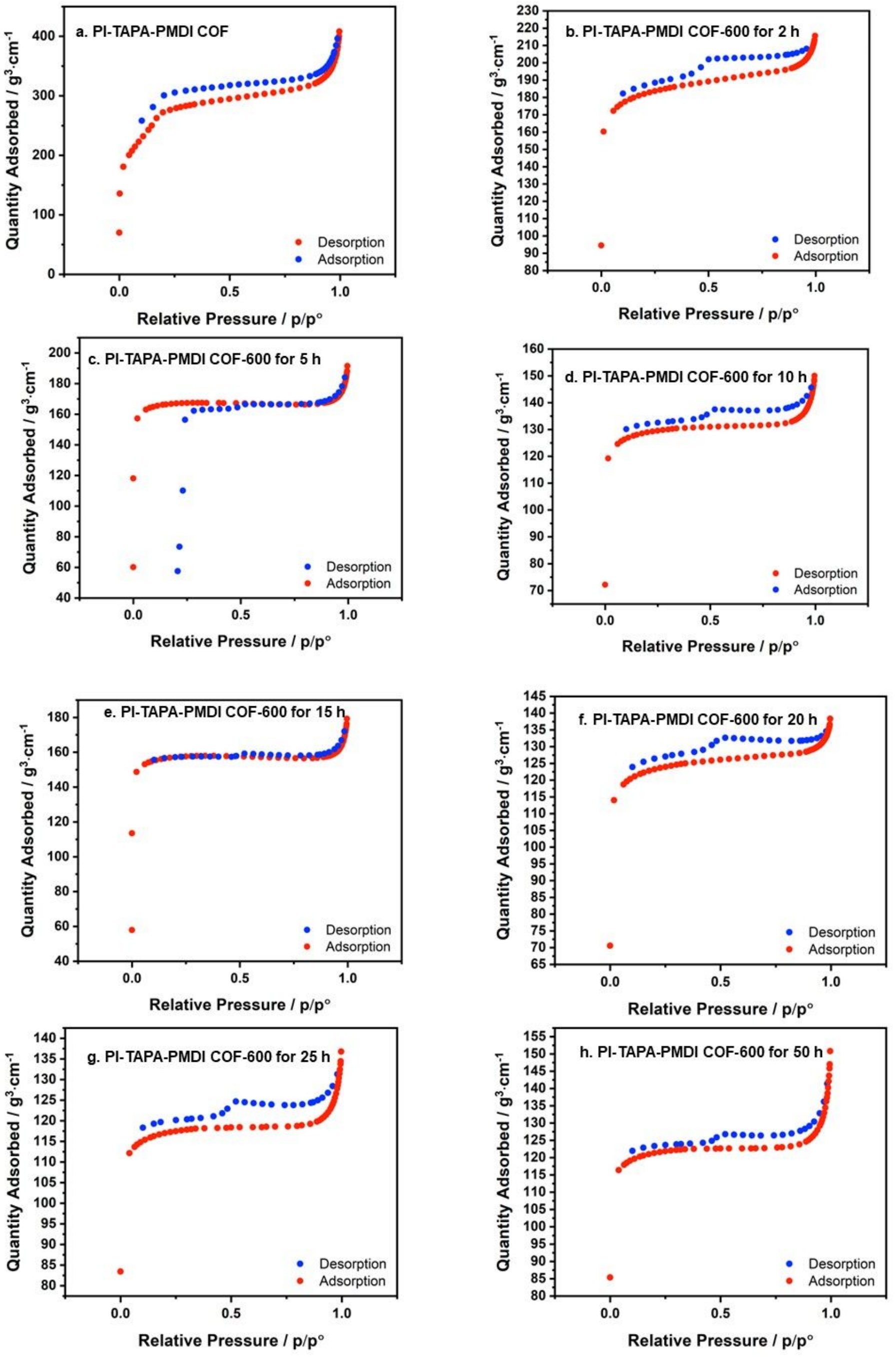

Figure S2. Nitrogen adsorption/desorption isotherms of a. PI-TAPA-PMDI COF, b. PI-TAPA-MDI COF-600 for 2h, c. PI-TAPA-PMDI COF-600 for 5h, d. PI-TAPA-PMDI COF-600 for 10h, e. PI-TAPA-PMDI COF-600 for 15h, f. PI-TAPA-PMDI COF-600 for 20h, g. PI-TAPA-PMDI COF-600 for 25h, h. PI-TAPA-PMDI COF-600 for 50h.

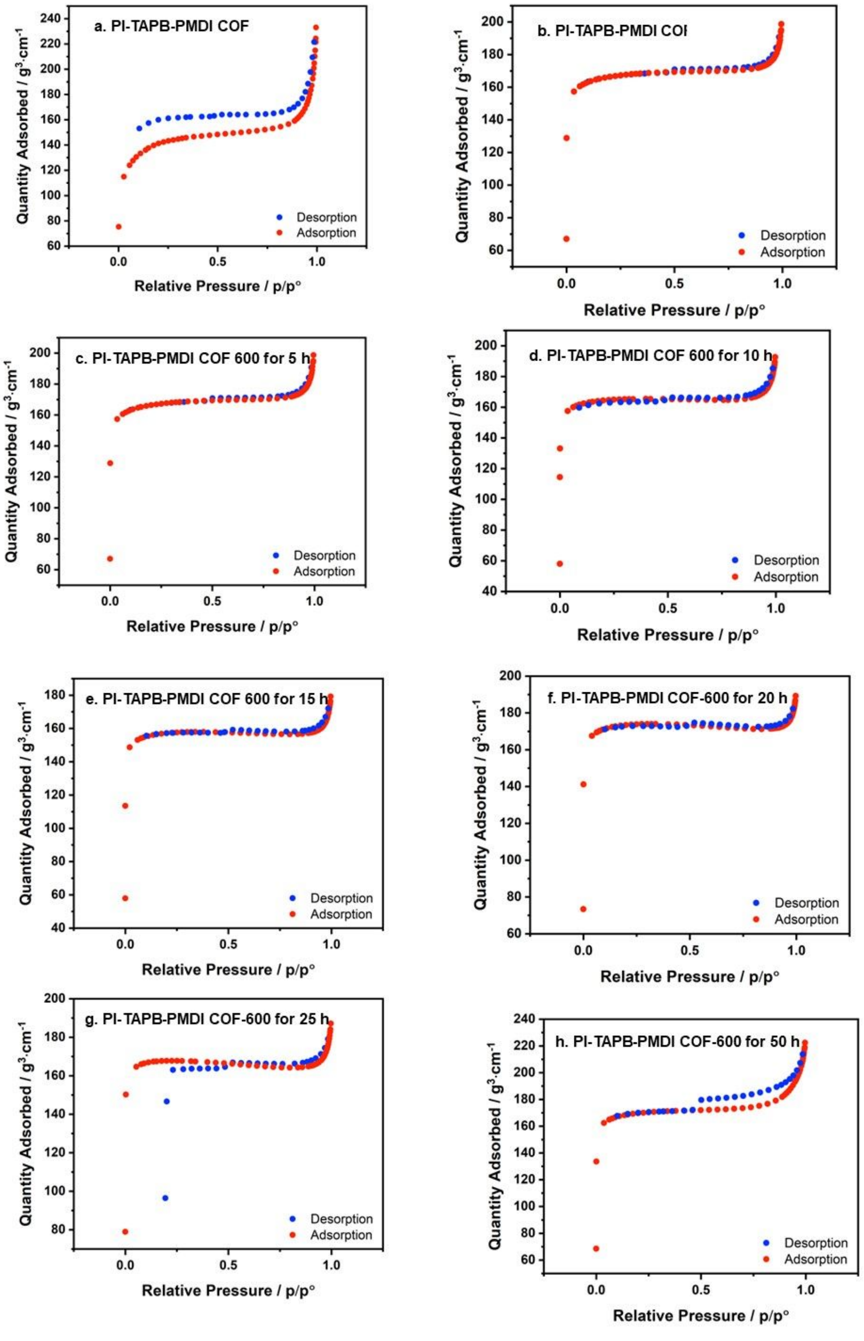

Figure S3. Nitrogen adsorption/desorption isotherms of a. PI-TAPB-PMDI COF, b. PI-TAPB-PMDI COF-600 for 2h, c. PI-TAPB-PMDI COF-600 for 5h, d. PI-TAPB-PMDI COF-600 for 10h, e. PI-TAPB-PMDI COF-600 for 15h, f. PI-TAPB-PMDI COF-600 for 20h, g. PI-TAPB-PMDI COF-600 for 25h, h. PI-TAPB-PMDI COF-600 for 50h.

Supporting Information

a. PI-TAPA-PMDI COF

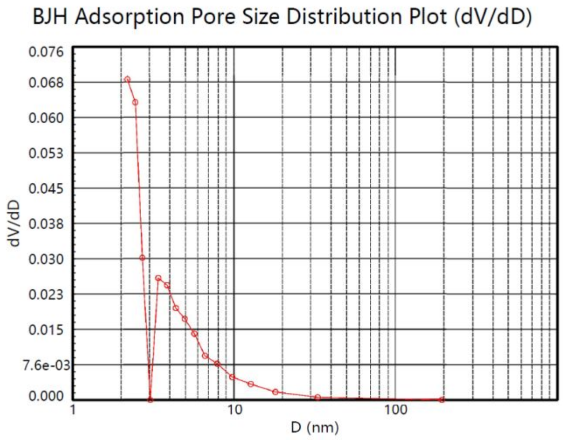

b. PI-TAPA-PMDI COF-600°C-2h

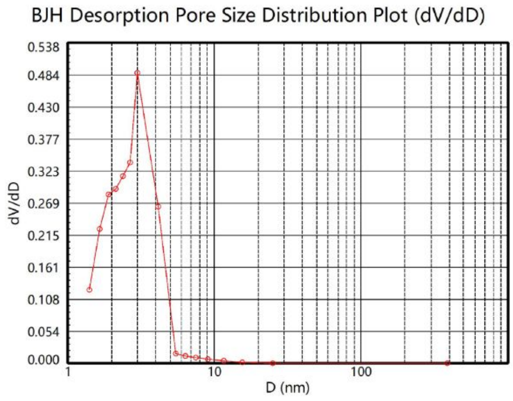

c. PI-TAPA-PMDI COF-600°C-5h

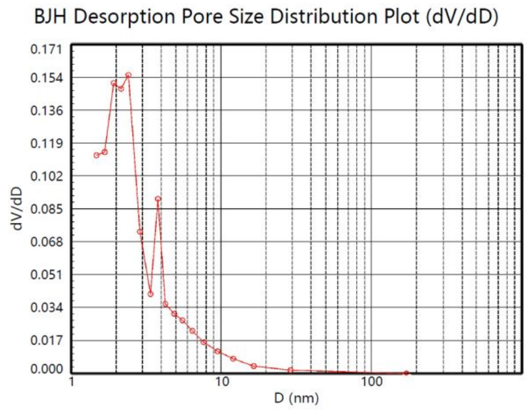

d. PI-TAPA-PMDI COF-600°C -10h

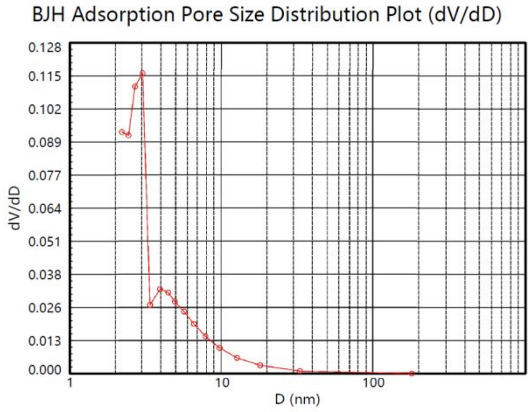

e. PI-TAPA-PMDI COF-600°C -15h

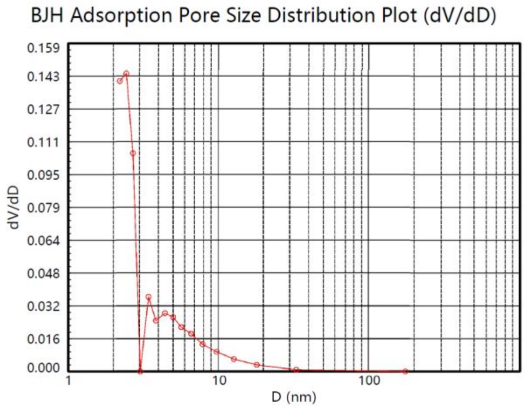

f. PI-TAPA-PMDI COF-600°C -20h

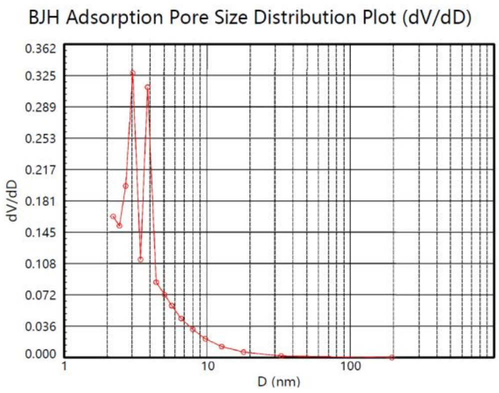

g. PI-TAPA-PMDI COF-600°C-25h

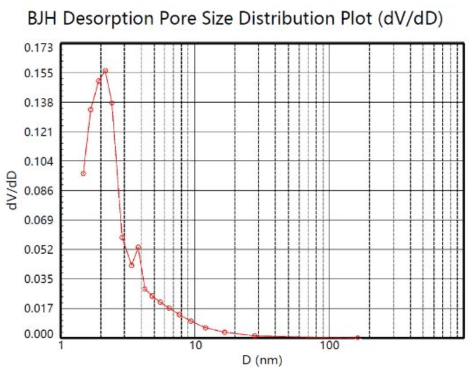

h. PI-TAPA-PMDA COF-600°C-50h

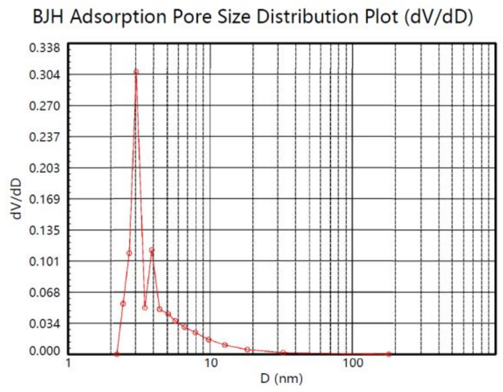

Figure S4. BJH Adsorption Pore Size Distribution Plots (dV/dD) of a. PI-TAPA-PMDI COF, b. PI-TAPA-PMDI COF-600°C for 2h, c. PI-TAPA-PMDI COF-600°C for 5h, d. PI-TAPA-PMDI COF-600°C for 10h, e. PI-TAPA-PMDI COF-600°C for 15h, f. PI-TAPA-PMDI COF-600°C for 20h, g. PI-TAPA-PMDI COF-600°C for 25h, h. PI-TAPA-PMDI COF-600°C for 50h.

a. PI-TAPB-PMDI COF

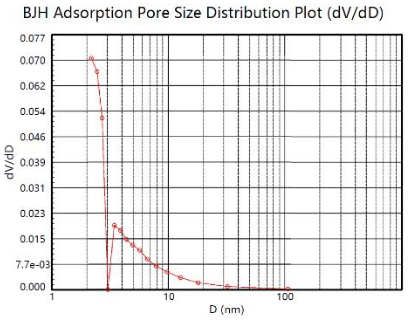

b. PI-TAPB-PMDI COF-600°C-2h

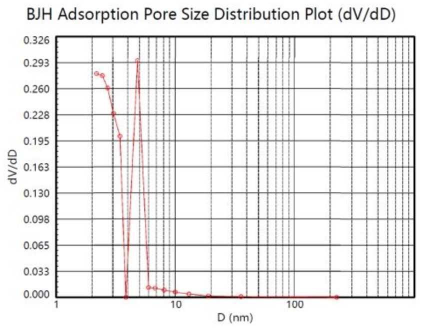

c. PI-TAPB-PMDI COF-600°C-5h

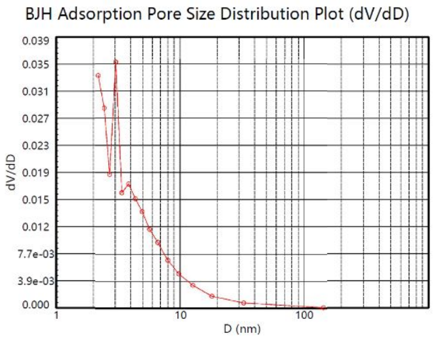

d. PI-TAPB-PMDI COF-600°C -10h

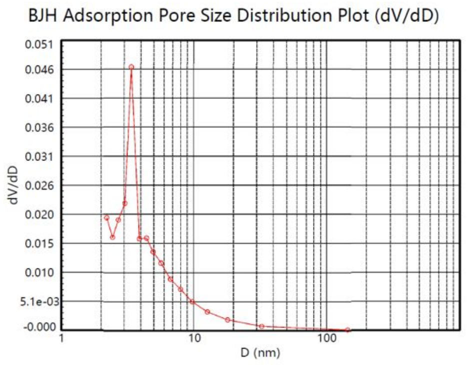

e. PI-TAPB-PMDI COF-600°C -15h

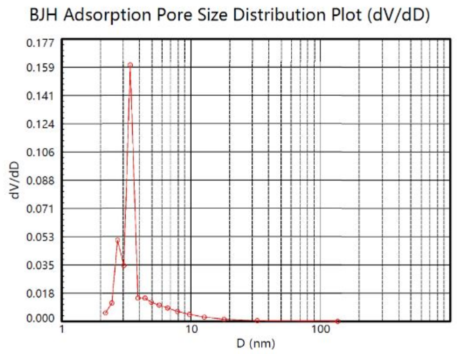

f. PI-TAPB-PMDI COF-600°C -20h

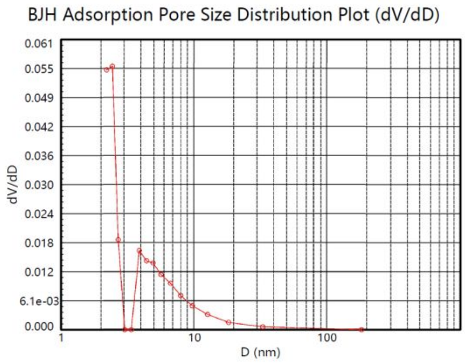

g. PI-TAPB-PMDI COF-600°C-25h

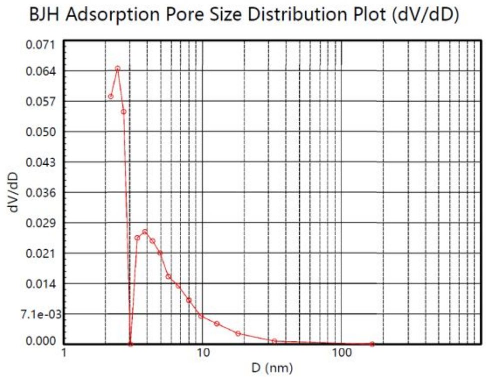

h. PI-TAPB-PMDA COF-600°C-50h

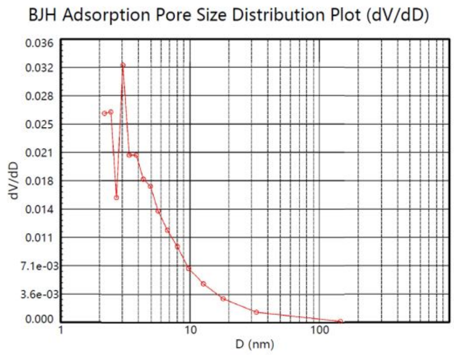

Figure S5. BJH Adsorption Pore Size Distribution Plots (dV/dD) of a. PI-TAPB-PMDI COF, b. PI-TAPB-PMDI COF-600°C for 2h, c. PI-TAPB-PMDI COF-600°C for 5h, d. PI-TAPB-PMDI COF-600°C for 10h, e. PI-TAPB-PMDI COF-600°C for 15h, f. PI-TAPB-PMDI COF-600°C for 20h, g. PI-TAPB-PMDI COF-600°C for 25h, h. PI-TAPB-PMDI COF-600°C for 50h.

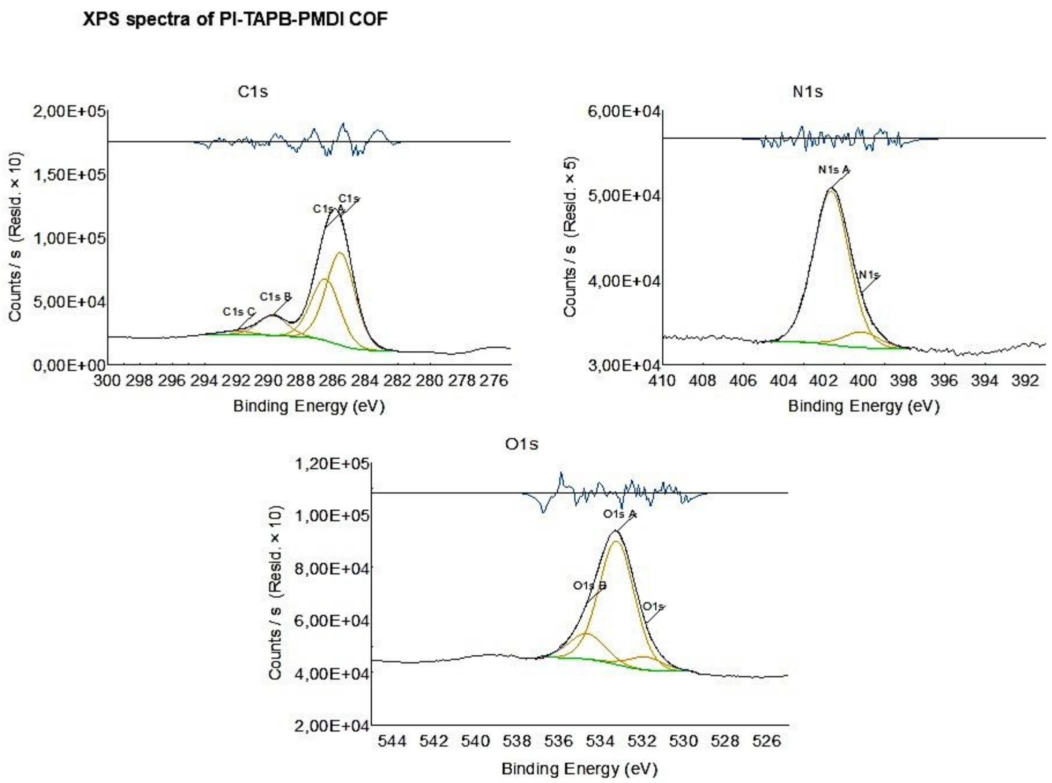

Figure S6. XPS Spectra of PI-TAPB-PMDI COF

Table S3. XPS peak assignments for PI-TAPB-PMDI COF

XPS Peak assignments of TAPB-PMDA COF											
Name	Peak BE	FWHM eV	Area (P) C	Atomic %	Q	SF	BE corr			Element	at.%
C1s	284.6	2.1	165514.9	43.0	1	0.25	285.0	C-C		C	82.6
C1s A	285.54	2.1	110835	28.8	1	0.25	285.9	C-O, C-N		O	11.0
C1s B	288.82	2.1	35709.75	9.3	1	0.25	289.2	C=O		N	6.4
C1s C	291.04	2.1	6061.81	1.6	1	0.25	291.4	COOH			100.0
O1s	532.3	1.99	10549.79	0.9	1	0.66	531.3	C=O			
O1s A	533.69	1.99	101863.3	8.4	1	0.66	532.7	C-O (aromatic)			
O1s B	535.12	1.99	21030.02	1.7	1	0.66	534.1	C-O (aliphatic)			
N1s	400.7	2.08	4196.8	0.6	1	0.42	399.6	C-NH+			
N1s A	402.2	2.08	40888.98	5.8	1	0.42	401.1	C-N, N=C			

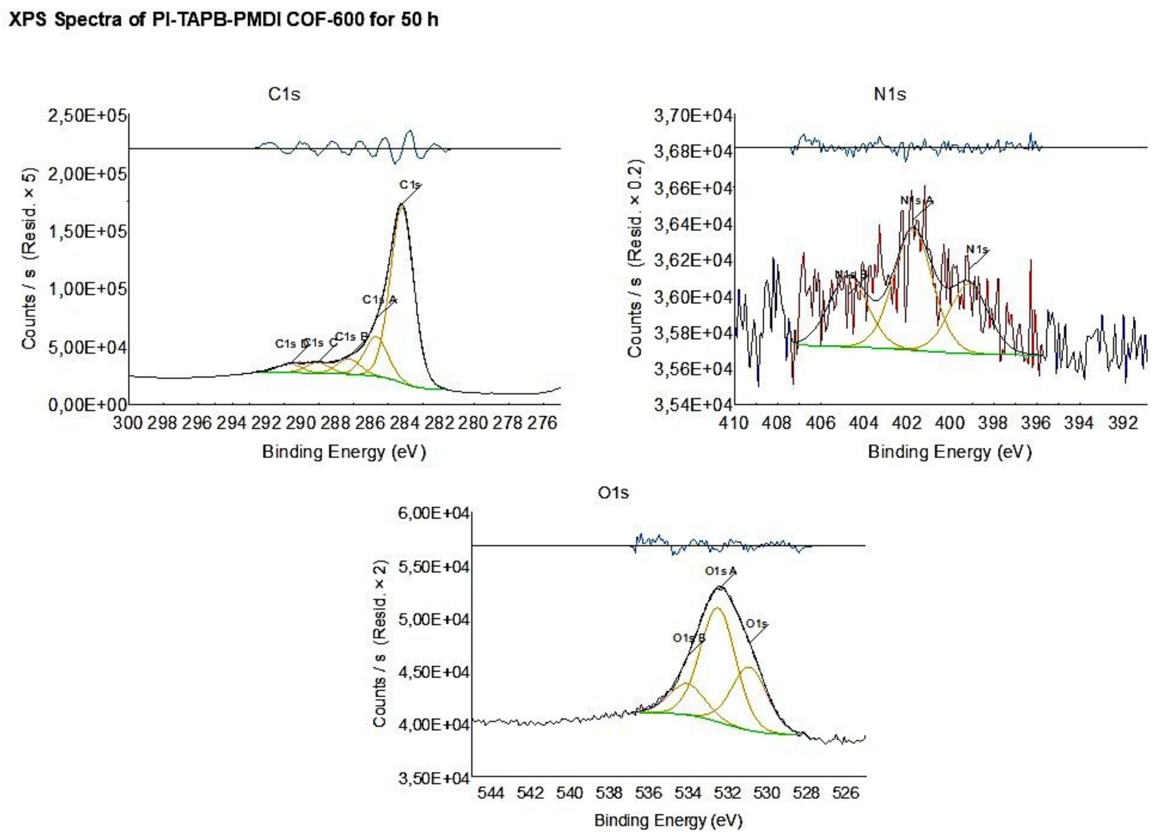

Figure S7. XPS Spectra of PI-TAPB-PMDI COF 600°C for 50h

Table S4. XPS peak assignments for PI-TAPB-PMDA COF 600°C for 50h

XPS Peak assignments of TAPB-PMDA COF at 50h heating (carbon)										
Name	Peak BE	FWHM eV	Area (P) C	Atomic %	Q	SF			Element	at.%
C1s	283.1	1.58	259861.4	67.3	1	0.25	sp2		C	95.8
C1s A	284.6	1.58	58703.53	15.2	1	0.25	sp3		O	3.7
C1s B	286.2	1.58	22882.08	5.9	1	0.25	C-O, C-N		N	0.5
C1s C	288.0	1.58	15862.64	4.1	1	0.25	C=O			100.0
C1s D	289.6	1.58	13114.25	3.4	1	0.25	COOH			
O1s	530.8	2.12	13696.78	1.1	1	0.66	C=O			
O1s A	532.4	2.12	24715.38	2.0	1	0.66	C-O			
O1s B	534.0	2.12	6767.63	0.6	1	0.66	C-O in aromatics			
N1s	397.0	2.12	878.52	0.1	1	0.42	C-N, N=C			
N1s A	399.5	2.12	1525.08	0.2	1	0.42	N-C=O			
N1s B	402.5	2.12	890.11	0.1	1	0.42	C-NH+			
